# Parliament and Publications

**Published:** 1910-12-17

**Authors:** 


### PARLIAMENT AND PUBLICATIONS.

### PUBLIC HEALTH IN SWAZILAND.

ACCORDING to the medical officer of Swaziland, eighty in-patients and 2,360 out-patients were treated in the hospital during the year ending March 31, 1910. Two more wards, an operating room and surgical instruments are required. Numerous cases of malarial fever occurred during the summer months; calomel and quinine were sent to all the assistant commissioners, but very little was asked for, the natives preferring their native doctors and native remedies. Europeans residing in the fever districts were informed that the Government was prepared to supply them with mosquito-proof wire-netting at cost price, but there were very few applicants. No fresh cases of leprosy were reported during the year. It is difficult to say how much syphilis there is among the Swazis, owing to the fact that they go to the native doctors for treatment. Native births and deaths are not registered, but the European death rate was 13.483 per 1,000.—"Colonial Reports: Swaziland," Cd. 4964, price 1½d.

